# Supplementary material for: Ultrafast photomechanical transduction through thermophoretic implosion
Source: Nat Commun. 2020 Jan 2;11:50. doi: 10.1038/s41467-019-13912-w (PMC6940389; doi:10.1038/s41467-019-13912-w)
Supplement: Supplementary file 1 — Supplementary Information [file 41467_2019_13912_MOESM1_ESM.pdf]

Supplementary information for:

Ultrafast photomechanical transduction through thermophoretic implosion

N. Kavokine, S. Zou, R. Liu, A. Niguès, B. Zou and L. Bocquet

1 Supplementary figures

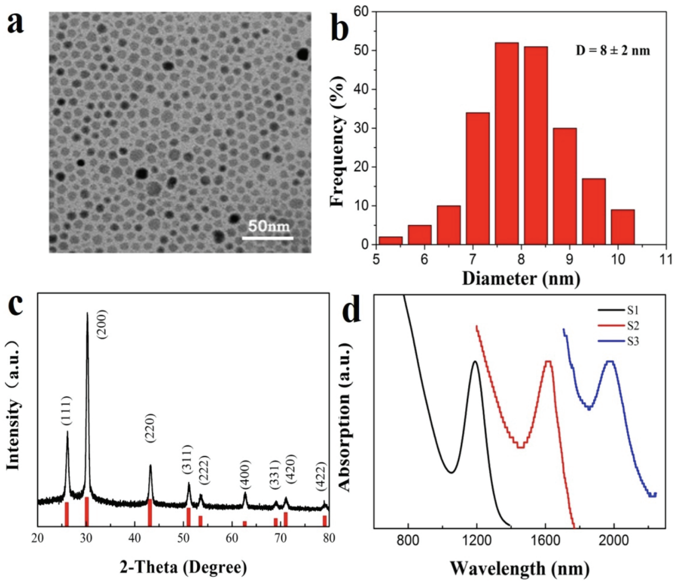

Supplementary figure 1: Characterisation of the PbS nanoparticles. **a.** TEM image of the PbS nanoparticles. **b.** Size distribution of the PbS nanoparticles as determined from TEM imaging. **c.** X ray diffraction spectrum of the PbS nanoparticles. **d.** Absorption spectra of PbS nanoparticles in cyclohexane. The three curves correspond to three samples with different average particle size.

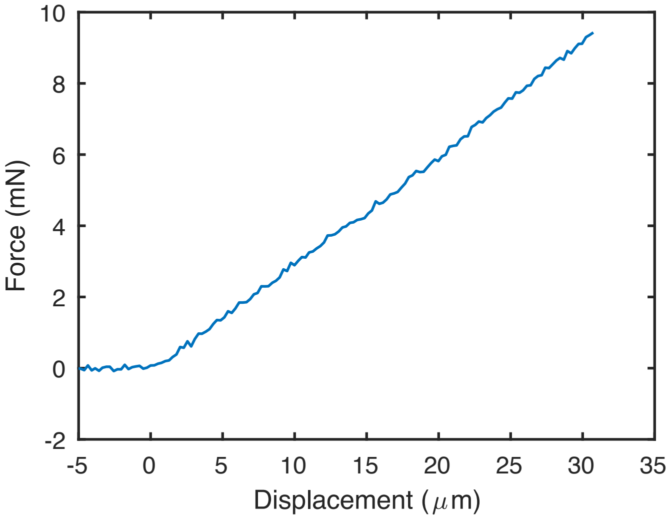

Supplementary figure 2: Force versus displacement curve for the cantilever used in the force measurements shown in fig. 2 of the main text. A Novatech F329 load cell was used for the calibration.

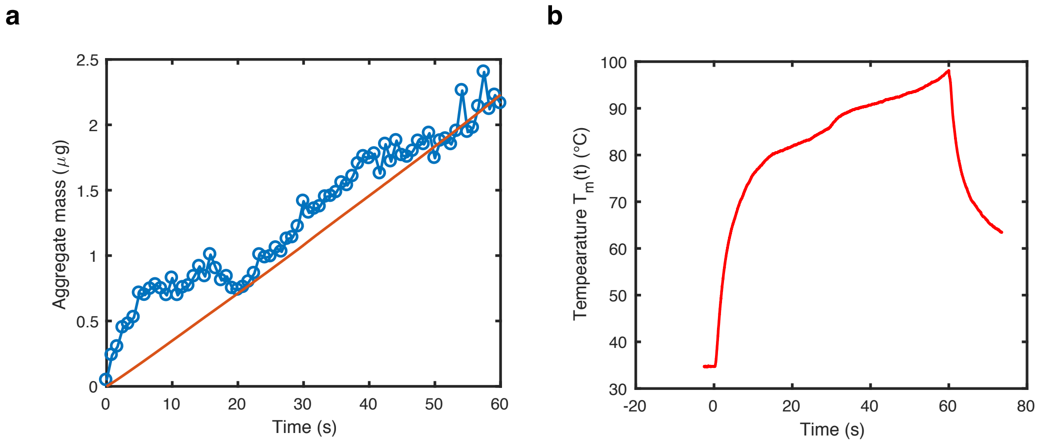

SUPPLEMENTARY FIGURE 3: Aggregate dynamics and determination of the Soret coefficient.

The blue curve in panel **a** shows the aggregate mass as a function of time, as determined from movie S4. The particle flux leading to the aggregate formation can be written as $\mathbf{j} = -\rho D S \nabla T - D \nabla \rho$ (with the same notations as in the main text). As a crude approximation, we may consider that the temperature field is only due to the aggregate, which behaves as a point heat source of slowly varying temperature $T_m(t)$. In the cylindrical geometry under consideration, this yields $\nabla T = T_m/r$. The steady state condition $\nabla \cdot \mathbf{j} = 0$ then reduces to $\Delta \rho = 0$. Imposing that far away from the aggregate the particle density is equal to the bulk density ρ_0, this yields a uniform density $\rho(r) = \rho_0$ in the whole solution. The aggregate mass as a function of the time t is then given by

$$M(t) = \pi h m \rho_0 D |S| T_m(t) t, \qquad (1)$$

where m is the mass of one particle and $h = 1$ mm the thickness of the spectroscopic cuvette. The red curve in panel **a** corresponds to eq. (1), with $T_m(t)$ extracted from the thermal imaging data (movie S5 and panel **b**), and $|S| = 4\ \mathrm{K}^{-1}$, which yields good agreement with the experimental data.

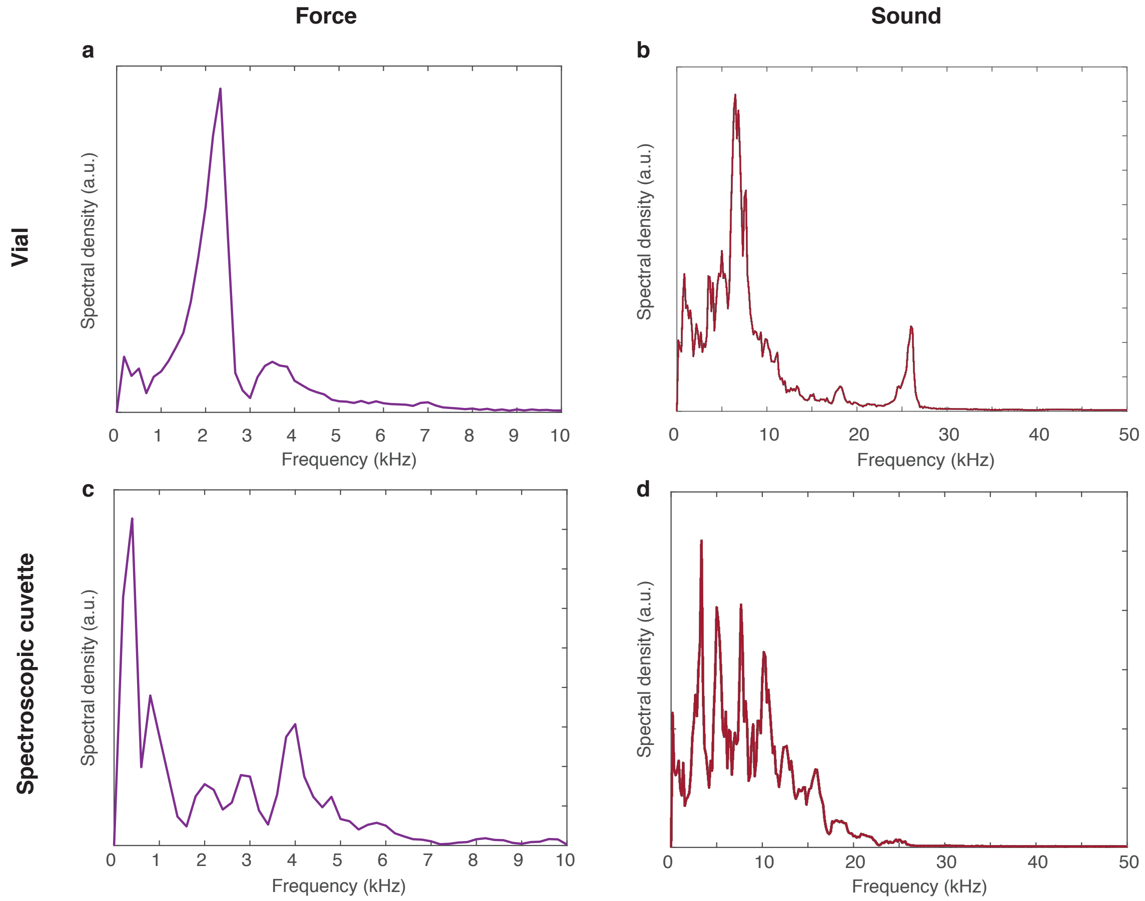

SUPPLEMENTARY FIGURE 4: Spectra of the force and sound spikes in the millilitre vial and in the one millimetre thick spectroscopic cuvette. The cuvette spectra were averaged over six spikes, while the vial spectra were averaged over several thousand spikes. The spectra are strongly affected by the nature of the container, hence the oscillations inside the spikes are likely to correspond to modes of the container's glass walls.

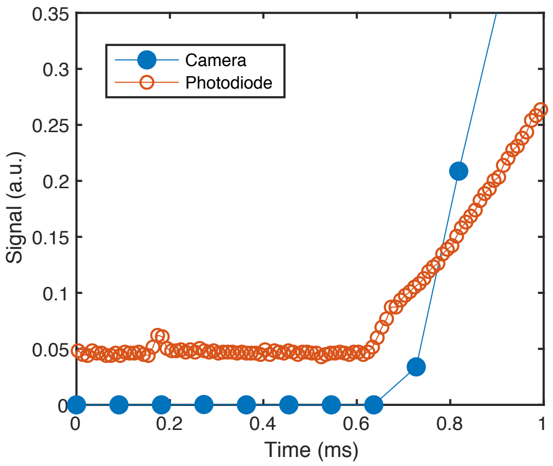

SUPPLEMENTARY FIGURE 5: Synchronisation test between the high-speed camera and the quadrant photodiode used for force measurement. The figure shows the signal on the camera and on the photodiode as a function of time when a light is suddenly switched on. The two signals appear to be synchronised down to one camera frame (90 μs).

2 Supplementary discussion

The dielectrophoretic force on a spherical particle of radius R_0 is [1]

$$F(r) = 2\pi R_0^3 \epsilon_s \frac{\epsilon_p - \epsilon_s}{\epsilon_p + 2\epsilon_s} \nabla(\epsilon_0 E(r)^2), \tag{2}$$

where ϵ_s and ϵ_p are the relative permittivities of the solvent and of the particle, respectively, considered here real since both materials in question are insulating; E is the electric field. The electromagnetic energy density corresponding to the diverging laser beam is $\epsilon_0 E(r)^2 \approx P/(2\pi r^2 c)$, where $P = 1.5$ W is the laser power. This yields, at a distance $r = 1$ mm from the fibre tip, $F \sim 10^{-23}$ N, which would lead to particle migration at a velocity $v \sim 10^{-13}$ m $\cdot$ s^{-1}, that is less than 1 nm per hour: this is incompatible with the experimentally observed timescales.

References

[1] Irimajiri, A., Hanai, T. & Inouye, A. A dielectric theory of "multi-stratified shell" model with its application to a lymphoma cell. *Journal of Theoretical Biology* **78**, 251–269 (1979).
