# Supplementary material for: Ultrafast photomechanical transduction through thermophoretic implosion
Source: Nat Commun. 2020 Jan 2;11:50. doi: 10.1038/s41467-019-13912-w (PMC6940389; doi:10.1038/s41467-019-13912-w)
Supplement: Supplementary file 3 — Description of Additional Supplementary Information [file 41467_2019_13912_MOESM3_ESM.docx]

**Description of Additional Supplementary Files**

**File Name**: Supplementary Movie 1
**Description:** A glass vial (height 33 mm with stopper, diameter 11 mm), filled with 1 mL of PbS nanoparticle solution in cyclohexane (4.8 wt %) rests on a flat aluminium surface. After 0.4 s (movie time) the laser is switched on, illuminating the vial through the fibre tip (on the left) at 2 W power. Upon laser illumination, the vial takes off from the substrate and "jumps" forward. One may notice a bubble forming at the illumination point as the vial takes off. Observation of the individual frames shows that the bubble is still growing when the vial has already taken off. The movie was recorded at 5400 frames per second and is slowed down 36 times.

**File Name:** Supplementary Movie 2
**Description:** A glass vial (height 33 mm with stopper, diameter 11 mm), filled with 1 mL of PbS nanoparticle solution in cyclohexane (4.8 wt %) rests on a flat aluminium surface. After 4 seconds the laser is switched on, illuminating the vial through the fibre tip (on the left) at 1.5 W power. Upon laser illumination, the vial jumps forward, falling out of range of the laser. The fibre tip is then moved towards the vial using a stepper motor, resulting in another propulsion event. The process is repeated several times, resulting in macroscopic motion of the vial at an average speed of about 1 mm/s. Two glass slides were placed on the aluminium surface so as to prevent the vial from shifting to the side. The movie is shown in real time.

**File Name:** Supplementary Movie 3
**Description:** (with sound) Observation of the bubble dynamics associated with the photomechanical effect. The PbS nanoparticle solution is imaged through the side of a 1 mm thick spectroscopic cuvette. The laser illuminated the solution through the short side of the cuvette (outside of the field of view, on the left). The laser is switched on at the beginning of the movie, at 2 W power. After 2 s movie time, one can observe a bubble explosively growing and then collapsing next to the cuvette wall, at the same time as a sound is emitted. Several explosive bubble formation events can be observed, until the process is "jammed" (after 28 s movie time), and a particle aggregate starts forming next to the cuvette wall. The movie was recorded at 5400 frames per second and is slowed down 164 times.

**File Name:** Supplementary Movie 4
**Description:** Observation of the particle aggregate dynamics on long timescales. The dynamics of the particle aggregate that forms after about a second of illumination in the spectroscopic cuvette are shown in real time.

**File Name:** Supplementary Movie 5
**Description:** Thermal camera movie showing the temperature distribution in the spectroscopic cuvette upon laser illumination at 2 W power.
